# Sex-specific lesion pattern of functional outcomes after stroke

**DOI:** 10.1093/braincomms/fcac020

**Published:** 2022-02-02

**Authors:** Anna K. Bonkhoff, Martin Bretzner, Sungmin Hong, Markus D. Schirmer, Alexander Cohen, Robert W. Regenhardt, Kathleen L. Donahue, Marco J. Nardin, Adrian V. Dalca, Anne-Katrin Giese, Mark R. Etherton, Brandon L. Hancock, Steven J. T. Mocking, Elissa C. McIntosh, John Attia, Oscar R. Benavente, Stephen Bevan, John W. Cole, Amanda Donatti, Christoph J. Griessenauer, Laura Heitsch, Lukas Holmegaard, Katarina Jood, Jordi Jimenez-Conde, Steven J. Kittner, Robin Lemmens, Christopher R. Levi, Caitrin W. McDonough, James F. Meschia, Chia-Ling Phuah, Arndt Rolfs, Stefan Ropele, Jonathan Rosand, Jaume Roquer, Tatjana Rundek, Ralph L. Sacco, Reinhold Schmidt, Pankaj Sharma, Agnieszka Slowik, Martin Söderholm, Alessandro Sousa, Tara M. Stanne, Daniel Strbian, Turgut Tatlisumak, Vincent Thijs, Achala Vagal, Johan Wasselius, Daniel Woo, Ramin Zand, Patrick F. McArdle, Bradford B. Worrall, Christina Jern, Arne G. Lindgren, Jane Maguire, Michael D. Fox, Danilo Bzdok, Ona Wu, Natalia S. Rost, MRI-GENIE and GISCOME Investigators and the International Stroke Genetics Consortium, Anna K. Bonkhoff, Martin Bretzner, Sungmin Hong, Markus D. Schirmer, Alexander Cohen, Robert W. Regenhardt, Kathleen L. Donahue, Marco J. Nardin, Adrian V. Dalca, Anne-Katrin Giese, Mark R. Etherton, Brandon L. Hancock, Steven J. T. Mocking, Elissa C. McIntosh, John Attia, Oscar R. Benavente, Stephen Bevan, John W. Cole, Amanda Donatti, Christoph J. Griessenauer, Laura Heitsch, Lukas Holmegaard, Katarina Jood, Jordi Jimenez-Conde, Steven J. Kittner, Robin Lemmens, Christopher R. Levi, Caitrin W. McDonough, James F. Meschia, Chia-Ling Phuah, Arndt Rolfs, Stefan Ropele, Jonathan Rosand, Jaume Roquer, Tatjana Rundek, Ralph L. Sacco, Reinhold Schmidt, Pankaj Sharma, Agnieszka Slowik, Martin Söderholm, Alessandro Sousa, Tara M. Stanne, Daniel Strbian, Turgut Tatlisumak, Vincent Thijs, Achala Vagal, Johan Wasselius, Daniel Woo, Ramin Zand, Patrick F. McArdle, Bradford B. Worrall, Christina Jern, Arne G. Lindgren, Jane Maguire, Michael D. Fox, Danilo Bzdok, Ona Wu, Natalia S. Rost

**Affiliations:** 1 J. Philip Kistler Stroke Research Center, Massachusetts General Hospital, Harvard Medical School, Boston, MA, USA; 2 Univ. Lille, Inserm, CHU Lille, U1171—LilNCog (JPARC)—Lille Neurosciences & Cognition, Lille F-59000, France; 3 Clinic for Neuroradiology, University Hospital Bonn, Bonn, Germany; 4 Center for Brain Circuit Therapeutics, Department of Neurology, Psychiatry, and Radiology, Brigham and Women’s Hospital, Harvard Medical School, Boston, MA, USA; 5 Department of Neurology, Boston Children’s Hospital, Harvard Medical School, Boston, MA, USA; 6 Computer Science and Artificial Intelligence Lab, Massachusetts Institute of Technology, Boston, MA, USA; 7 Athinoula A. Martinos Center for Biomedical Imaging, Department of Radiology, Massachusetts General Hospital, Charlestown, MA, USA; 8 Department of Neurology, University Medical Center Hamburg-Eppendorf, Hamburg, Germany; 9 Hunter Medical Research Institute, Newcastle, NSW, Australia; 10 School of Medicine and Public Health, University of Newcastle, Newcastle, NSW, Australia; 11 Department of Medicine, Division of Neurology, University of British Columbia, Vancouver, BC, Canada; 12 School of Life Sciences, University of Lincoln, Lincoln, UK; 13 Department of Neurology, University of Maryland School of Medicine and Veterans Affairs Maryland Health Care System, Baltimore, MD, USA; 14 School of Medical Sciences, University of Campinas (UNICAMP) and the Brazilian Institute of Neuroscience and Neurotechnology (BRAINN), Campinas, SP, Brazil; 15 Department of Neurosurgery, Geisinger, Danville, PA, USA; 16 Research Institute of Neurointervention, Paracelsus Medical University, Salzburg, Austria; 17 Department of Emergency Medicine, Washington University School of Medicine, St Louis, MO, USA; 18 Department of Neurology, Washington University School of Medicine & Barnes-Jewish Hospital, St Louis, MO, USA; 19 Department of Clinical Neuroscience, Institute of Neuroscience and Physiology, Sahlgrenska Academy, University of Gothenburg, Gothenburg, Sweden; 20 Department of Neurology, Sahlgrenska University Hospital, Gothenburg, Sweden; 21 Department of Neurology, Neurovascular Research Group (NEUVAS), IMIM-Hospital del Mar (Institut Hospital del Mar d’Investigacions Mèdiques), Universitat Autonoma de Barcelona, Barcelona, Spain; 22 Department of Neurosciences, Experimental Neurology and Leuven Research Institute for Neuroscience and Disease (LIND), KU Leuven—University of Leuven, Leuven, Belgium; 23 Department of Neurology, VIB, Vesalius Research Center, Laboratory of Neurobiology, University Hospitals Leuven, Leuven, Belgium; 24 Department of Neurology, John Hunter Hospital, Newcastle, NSW, Australia; 25 Department of Pharmacotherapy and Translational Research and Center for Pharmacogenomics, University of Florida, Gainesville, FL, USA; 26 Department of Neurology, Mayo Clinic, Jacksonville, FL, USA; 27 Centogene AG, Rostock, Germany; 28 Department of Neurology, Clinical Division of Neurogeriatrics, Medical University Graz, Graz, Austria; 29 Center for Genomic Medicine, Massachusetts General Hospital, Boston, MA, USA; 30 Department of Neurology and Evelyn F. McKnight Brain Institute, Miller School of Medicine, University of Miami, Miami, FL, USA; 31 Institute of Cardiovascular Research Royal Holloway, University of London (ICR2UL), London, UK; 32 St Peter’s and Ashford Hospital, Egham, UK; 33 Department of Neurology, Jagiellonian University Medical College, Krakow, Poland; 34 Department of Clinical Sciences Malmö, Lund University, Lund, Sweden; 35 Department of Neurology, Skåne University Hospital, Lund and Malmö, Malmo, Sweden; 36 Department of Laboratory Medicine, Institute of Biomedicine, The Sahlgrenska Academy, University of Gothenburg, Gothenburg, Sweden; 37 Department of Neurology, Helsinki University Hospital and University of Helsinki, Helsinki, Finland; 38 Department of Clinical Neuroscience, Institute of Neuroscience and Physiology, Sahlgrenska Academy at University of Gothenburg, Gothenburg, Sweden; 39 Stroke Division, Florey Institute of Neuroscience and Mental Health, Heidelberg, Australia; 40 Department of Neurology, Austin Health, Heidelberg, Australia; 41 Department of Radiology, University of Cincinnati College of Medicine, Cincinnati, OH, USA; 42 Department of Clinical Sciences Lund, Radiology, Lund University, Lund, Sweden; 43 Department of Radiology, Neuroradiology, Skåne University Hospital, Lund, Sweden; 44 Department of Neurology and Rehabilitation Medicine, University of Cincinnati College of Medicine, Cincinnati, OH, USA; 45 Department of Neurology, Geisinger, Danville, PA, USA; 46 Division of Endocrinology, Diabetes and Nutrition, Department of Medicine, University of Maryland School of Medicine, Baltimore, MD, USA; 47 Department of Neurology, University of Virginia, Charlottesville, VA, USA; 48 Department of Public Health Sciences, University of Virginia, Charlottesville, VA, USA; 49 Department of Clinical Genetics and Genomics, Sahlgrenska University Hospital, Gothenburg, Sweden; 50 Department of Neurology, Skåne University Hospital, Lund, Sweden; 51 Department of Clinical Sciences Lund, Neurology, Lund University, Lund, Sweden; 52 University of Technology Sydney, Sydney, Australia; 53 Department of Biomedical Engineering, McConnell Brain Imaging Centre, Montreal Neurological Institute, Faculty of Medicine, School of Computer Science, McGill University, Montreal, Canada; 54 Mila—Quebec Artificial Intelligence Institute, Montreal, Canada

**Keywords:** acute ischaemic stroke, functional outcomes, sex differences, lesion patterns, Bayesian hierarchical modelling

## Abstract

Stroke represents a considerable burden of disease for both men and women. However, a growing body of literature suggests clinically relevant sex differences in the underlying causes, presentations and outcomes of acute ischaemic stroke. In a recent study, we reported sex divergences in lesion topographies: specific to women, acute stroke severity was linked to lesions in the left-hemispheric posterior circulation. We here determined whether these sex-specific brain manifestations also affect long-term outcomes. We relied on 822 acute ischaemic patients [age: 64.7 (15.0) years, 39% women] originating from the multi-centre MRI-GENIE study to model unfavourable outcomes (modified Rankin Scale >2) based on acute neuroimaging data in a Bayesian hierarchical framework. Lesions encompassing bilateral subcortical nuclei and left-lateralized regions in proximity to the insula explained outcomes across men and women (area under the curve = 0.81). A pattern of left-hemispheric posterior circulation brain regions, combining left hippocampus, precuneus, fusiform and lingual gyrus, occipital pole and latero-occipital cortex, showed a substantially higher relevance in explaining functional outcomes in women compared to men [mean difference of Bayesian posterior distributions (men – women) = −0.295 (90% highest posterior density interval = −0.556 to −0.068)]. Once validated in prospective studies, our findings may motivate a sex-specific approach to clinical stroke management and hold the promise of enhancing outcomes on a population level.

## Teaser

Relying on neuroimaging and clinical data of 822 acute stroke patients, Bonkhoff *et al.* report substantially more detrimental effects of lesions in left-hemispheric posterior circulation regions on functional outcomes in women compared to men. These findings may motivate a sex-specific clinical stroke management to improve outcomes in the longer term.

## Graphical Abstract

**Graphical Abstract:**
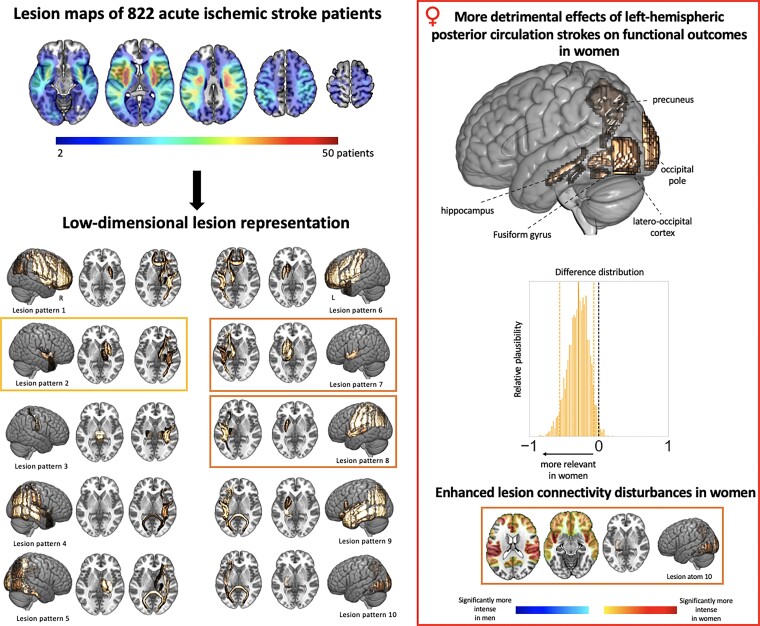

## Introduction

Stroke results in a considerable burden of disease for both men and women. Converging evidence, however, underscores the relevant influence of biological sex on the idiosyncrasies of cause, presentation and outcome of acute ischaemic stroke (AIS).^1^ Of note, women appear to feature a higher AIS severity upon admission that cannot be satisfactorily explained by any sociodemographic or clinical factors, such as age and cardioembolic stroke subtype.^2^ In particular, this higher symptom load emerges despite comparable lesion characteristics,^3,4^ consistent with the possibility that male and female brains might react differently to ischaemia-induced lesions. More concretely, we recently noted distinct sex divergences in stroke lesion topographies: stroke severity in both men and women was explained by lesions affecting presumed bilateral motor regions and left-lateralized language regions. However, only in women, stroke severity was additionally explained by lesions extending to the posterior circulation of the *left* hemisphere, rendering female-specific lesion pattern more wide-spread. Whether these sex-specific effects have an impact on acute stroke symptoms only, or have a long-lasting character, is currently unknown. The aim of the present study was to address these gaps in our understanding of sex-specific lesion effects on long-term stroke outcomes.

## Materials and methods

### AIS patient sample

All MRI-Genetics Interface Exploration (MRI-GENIE)^5^ AIS patients with available high-quality diffusion-weighted imaging (DWI)-derived lesion segmentations^6^ and 3 months (60–190 days) modified Rankin Scale (mRS) data were included in this complete case study (c.f., Supplementary materials for a sample size calculation). MRI-GENIE is a large international collaboration, built upon the infrastructure of the Stroke Generics Network (SiGN).^7^ It assembled sociodemographic, clinical, neuroimaging and genetic data of ∼3300 AIS patients, with the primary aim to facilitate genetic analyses of neuroimaging phenotypes as derived from clinical scans.^5^ While MRI-GENIE merged data from 12 international sites overall, analyses here primarily relied on five individual studies that could additionally share functional outcomes (c.f., Supplementary Table 1 for study sizes and characteristics). Subjects gave written informed consent in accordance with the Declaration of Helsinki. The study protocol was approved by the Massachusetts General Hospital’s Institutional Review Board.

### Stroke lesion representation derivation

DWI data and respective automatically DWI-derived lesions were non-linearly spatially normalized (c.f., Wu *et al*.^6^ for details on lesion segmentation and spatial normalization and Supplementary materials for imaging parameters). Spatially normalized DWI data and lesion segmentations were carefully quality controlled by two experienced raters (A.K.B. and M.B.). Lesion data were parsed according to 94 cortical regions, 15 subcortical regions^8^ and 20 white matter tracts,^9^ i.e. the number of lesioned voxels per atlas-defined brain region was computed. Non-negative matrix factorization (NMF) was used to reduce the collection of 129 atlas-based region and tract lesion measures to 10 unique lesion patterns. The number 10 was chosen in line with previous work^4,10–12^ and further motivated by achieving a balance between faithful representation of the high-dimensional input space and mitigation of the risk of overfitting.

### Statistical analyses: modelling unfavourable functional outcomes

Unfavourable functional outcome (mRS > 2) was modelled via the Bayesian hierarchical logistic regression. The 10 lesion patterns were incorporated as input variables with hierarchical priors capturing the biological sex of participants (as indicated in medical records), that is, one for female and one for male participants. The covariates age, age-squared, sex, cardiovascular risk factors (history of hypertension, atrial fibrillation, diabetes, ischaemic heart disease, prior stroke and smoking) and total DWI-derived lesion volume were included in the model (c.f., Supplementary materials for full model specifications).^4,10,11^ We did not incorporate admission stroke severity as an input variable, given that it likely reflects the extent and location of brain injury. Samples from the Bayesian posterior parameter distribution were drawn by employing the No U-Turn Sampler (NUTS), a type of Monte Carlo Markov Chain algorithm (setting: draws = 5000).^13^ The model classification performance of unfavourable outcomes was measured by the area under the receiver operating characteristic curve (AUC). We first assessed similarities between male- and female-specific lesion pattern effects and determined those lesion patterns with posterior distributions substantially differing from zero in both men and women. Differences between hierarchically estimated female and male lesion pattern effects were then evaluated via subtracting the corresponding posterior parameter distributions. As in previous work,^4^ we presumed substantial lesion pattern effects or sex differences if the resulting posterior (difference) distributions did not include zero in the 90% highest probability density interval (HPDI).

### Sensitivity analyses

We evaluated whether there were any general sex differences in lesion anatomy, i.e. the total DWI-derived lesion volume, as well as parcel-wise lesion volumes and frequencies in how often parcels were affected.

There was a slight imbalance in the men:women ratio in our sample. Altogether, there were more male patients experiencing a favourable long-term outcome. To ensure that results were not skewed by varying frequencies of unfavourable functional outcomes in women and men, we repeated analyses after downsampling the majority class, i.e. matching the occurrence of favourable outcomes in the groups of men and women. More specifically, we downsampled the larger group of men with favourable outcomes (*n* = 386) to the size of the smaller group of women with favourable outcomes (*n* = 208). That is, we randomly chose 208 of the 386 men with favourable outcomes and repeated this process 20 times. Unfavourable long-term outcomes were present in the same number of male and female patients (*n* = 114 each). Afterwards, we repeatedly conducted the Bayesian logistic regression analyses.

### Lesion network mapping analyses

Finally, we employed lesion network mapping^14^ (LNM) aiming to uncover links between lesion patterns and sex-specific lesion disconnection profiles. To achieve this goal, we implemented two main changes compared to classic LNM analyses^15,16^: first, instead of computing lesion network maps for lesions of individual patients, we utilized the 10 prototypical lesion patterns as regions of interest for the estimation of whole-brain lesion connectivity. We created 100 variations of each of the 10 lesion patterns by sampling a random number and collection of lesion pattern-affiliated brain regions (a brain region was considered affiliated, if the NMF-weight exceeded 0.05). Second, we relied on male- and female-specific normative connectomes, i.e. connectomes that were based on data of only male or female healthy participants (*n* = 346 each). Consequently, we obtained *two* LNM exemplars, a male- and a female-specific one, for each lesion pattern (conventionally: one LNM exemplar based on connectomes of *n* = 1000 healthy male *and* female participants).^14^ The preprocessing of sex-specific resting-state fMRI data itself was performed as previously described. Global signal regression was included. After estimating the whole-brain voxel-wise lesion connectivity and Fisher’s *z*-transformation, we summarized the positive and negative *t*-values, i.e. the ‘intensity’ of lesion connectivity, within Yeo/Schaefer-defined cortical networks^17,18^ (7 per hemisphere, therefore 14 in total; visual, somatomotor, dorsal attention, ventral attention, limbic, frontoparietal, default mode network). The Yeo–Schaefer atlas was here chosen to match the functional data at hand, as it was derived from functional data, in contrast to the Harvard–Oxford (HO)^8^ or JHU atlases,^9^ which are based on structural data. Eventually, we compared network-averaged intensities between the male- and female-connectome-based maps for each lesion pattern (two-sided *t*-tests, level of significance *P* < 0.05, false discovery rate-corrected for 14 hemisphere-specific network values for 10 lesion patterns).

### Code and data availability

The authors agree to make data available to researchers for the explicit purposes of reproducing the here stated results, pending the permission for data sharing by the Massachusetts General Hospital’s institutional review board. The HO and JHU DTI-based white matter atlases are openly available at https://fsl.fmrib.ox.ac.uk/fsl/fslwiki/Atlases. Analyses were implemented in the Python 3.7 (mainly employing the packages nilearn^19^ and pymc3^20^). Exemplary code is available under: https://github.com/AnnaBonkhoff/BMH_functional_outcomes_sex_differences.

## Results

### Prediction of unfavourable functional outcomes across men and women

**Figure 1:**
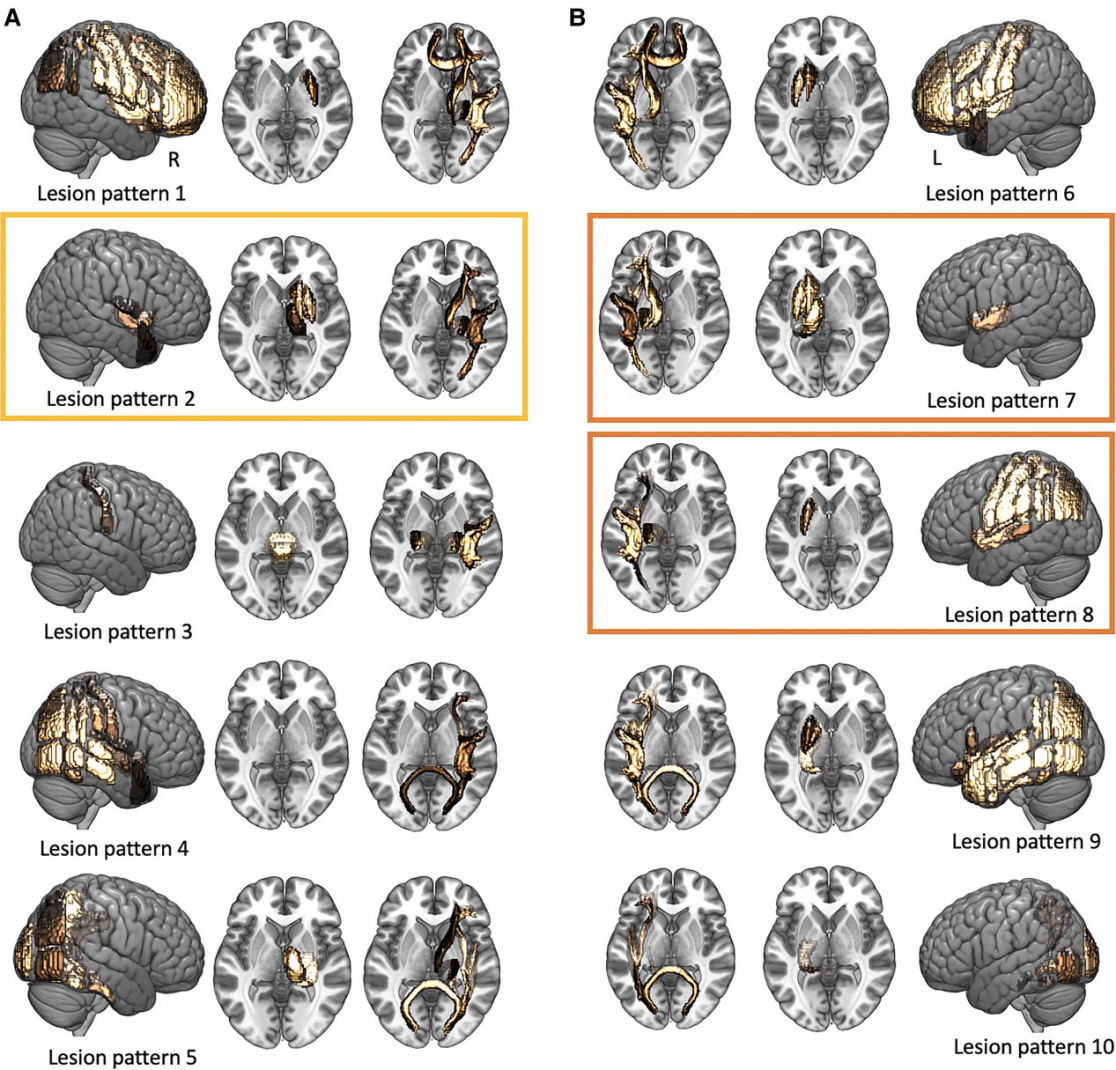
**Anatomically plausible, parsimonious representation of stroke lesions.** Ten unique, archetypical stroke lesion patterns were derived via an unsupervised pattern-discovery framework. Lesion pattern represented predominantly right-hemispheric stroke (**A**) and left-hemispheric stroke (**B**) with varying emphases on cortical–subcortical and anterior–medial–posterior regions. Three lesion patterns, framed in yellow for right-hemispheric stroke and in orange for left-hemispheric stroke, had a high relevance in explaining unfavourable functional outcome 3 months after stroke for both men and women. This relevance was discernible from their Bayesian posterior distributions that did not substantially overlap with zero (i.e. their 90% credibility intervals did not include zero).

**Figure 2:**
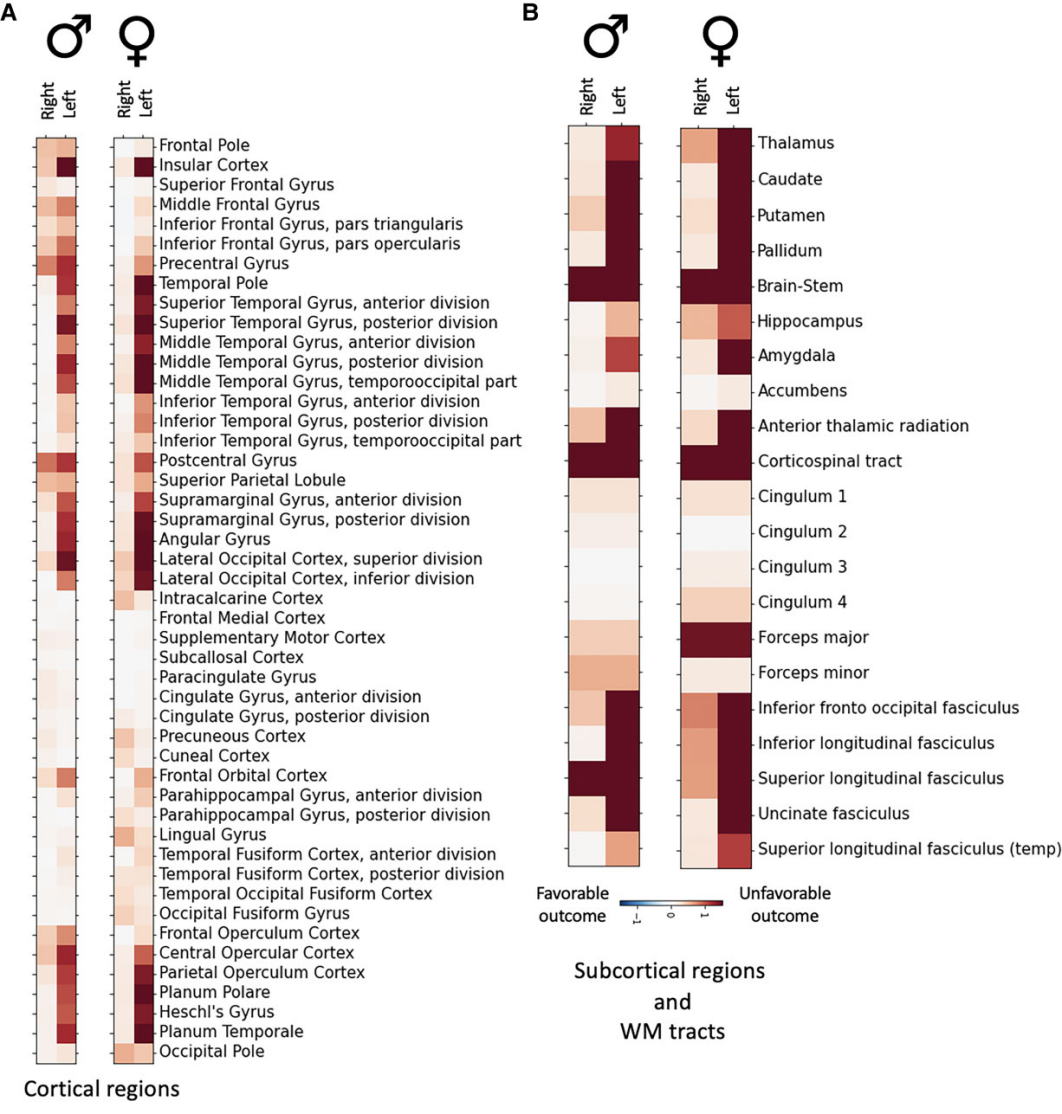
**Individual brain regions explaining unfavourable functional outcomes.** Characteristic constellations of cortical (**A**) and subcortical brain regions, as well as white matter tracts (**B**) emerged that explained unfavourable outcomes ∼3 months after stroke in 500 male and 322 female stroke patients. Lesions in the left hemisphere were more strongly associated with unfavourable long-term outcomes than lesions in the right hemisphere for both men and women. Particularly relevant regions comprised left pre- and post-central, insular and opercular cortex, superior and middle temporal gyri, supramarginal and angular gyrus and lateral occipital cortex.

A total of 822 AIS patients were recruited to model unfavourable long-term outcomes [age: 64.7 (15.0) years, 39% women, 28% unfavourable functional outcome, c.f., Table 1 for further clinical characteristics and Supplementary Fig. 1 for a lesion overlap]. Individual atlas-parcellated lesions were represented in five anatomically plausible lesion patterns per hemisphere, with varying emphases on subcortical to cortical and anterior to posterior regions (Fig. 1). Three of these 10 unique patterns explained considerable variation in unfavourable outcomes concurrently in both male and female patients (AUC = 0.81). The relevance in explaining unfavourable outcomes across men and women of these three spatial lesion patterns were indicated by 90% posterior intervals of Bayesian parameter distributions excluding zero for both biological sexes (men: Lesion pattern #2: posterior mean: 0.20, 90% HPDI: 0.04–0.35; Lesion pattern #7: posterior mean: 0.57, 90% HPDI: 0.11–1.07; Lesion pattern #8: 0.37, 90% HPDI: 0.01–0.72; women: Lesion pattern #2: posterior mean: 0.21, 90% HPDI: 0.07–0.35; Lesion pattern #7: posterior mean: 0.76, 90% HPDI: 0.13–1.25; Lesion pattern #8: 0.39, 90% HPDI: 0.07–0.72; Supplementary Figs 2 and 3). Mainly implicated brain regions in these three patterns were bilateral subcortical grey matter nuclei and left-lateralized regions in proximity to the insula (Figs 1 and 2).

**Table 1:** Patient characteristics

	All participants (*n* = 822)	Male participants (*n* = 500)	Female participants (*n* = 332)	Statistical comparison of male and female participants
Age, years	64.7 (15.0)	63.9 (14.2)	65.8 (16.2)	*P* = 0.07
Sex	39.2%	–	–	—
Unfavourable outcome (mRS > 2)	27.7%	22.8%	35.4%	*P* = 0.0001
Normalized DWI-derived stroke lesion volume (ml, median, interquartile range)	3.3 (0–12.8)	2.9 (0–11.3)	3.8 (0–17.8)	*P* = 0.28
Hypertension	64.1%	63.0%	65.9%	*P* = 0.41
Diabetes mellitus Type 2	21.8%	23.0%	19.9%	*P* = 0.30
Atrial fibrillation	16.8%	14.6%	20.2%	*P* = 0.04
Coronary artery disease	18.4%	21.8%	13.0%	*P* = 0.002
Smoking	55.0%	61.0%	45.7%	*P* < 0.0001
Prior stroke	9.7%	9.4%	10.2%	*P* = 0.72

Mean values and standard deviation, unless otherwise noted. Characteristics of men and women were compared either via two-sample *t*-tests or two-sided Fisher’s exact tests as appropriate. Significantly more women than men experienced unfavourable outcomes obtained ∼3 months post-stroke (women: 35.4% versus men: 22.8%). In view of this difference in our main outcome, we performed additional downsampling analyses, in which we repeatedly contrasted samples of male and female patients with the same ratios of favourable to unfavourable outcomes.

### Sex-specific prediction of unfavourable functional outcomes

**Figure 3:**
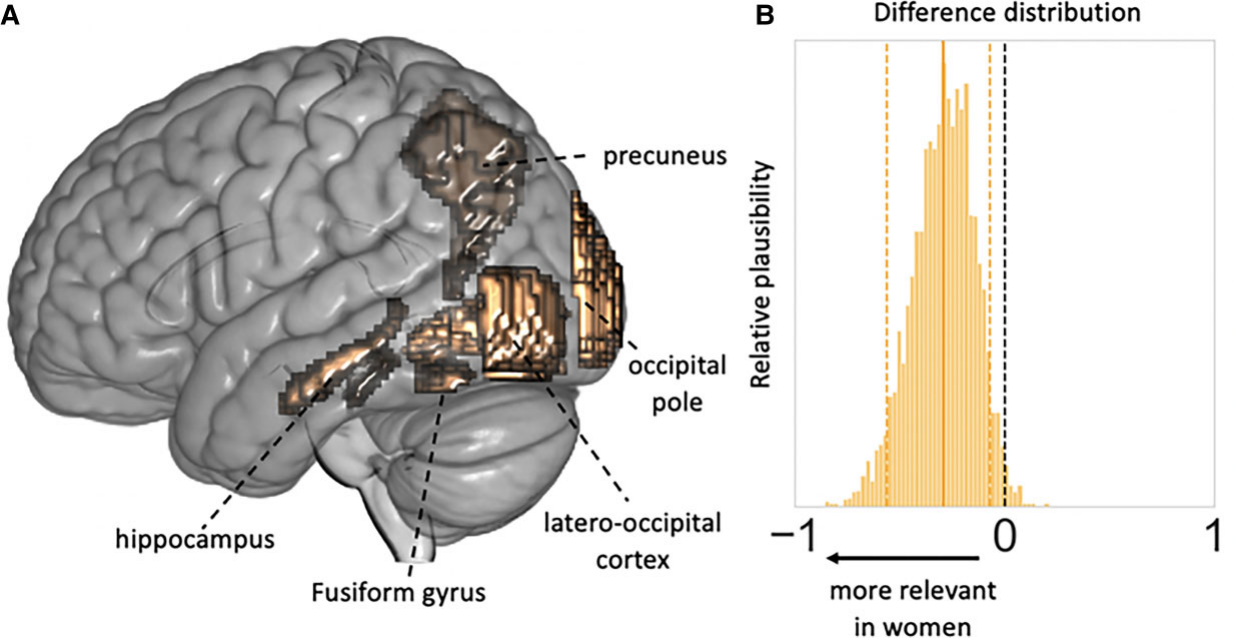
**Sex-specific effects relating to left-hemispheric posterior circulation lesion pattern**. (**A**) Lesion pattern #10 represented left-hemispheric lesions in the presumed posterior circulation, combining the hippocampus, precuneus, fusiform and lingual gyrus, latero-occipital cortex and occipital pole. (**B**) The difference distribution of Bayesian posteriors for the male- and female-specific expression of Lesion pattern #10 indicated a substantially higher relevance in explaining unfavourable functional outcome specifically in women (mean of the difference posterior distribution = −0.295, HPDI of the posterior distribution covering 90% certainty = −0.556 to −0.068).

Next, we compared the relevance of lesion patterns in men versus in women via contrasting their respective sex-specific lesion pattern posterior distributions. We here observed one predominant sex difference: the left-hemispheric lesion pattern of posterior circulation brain regions was assigned a substantially higher relevance in explaining unfavourable functional outcome specifically in women [mean difference (men – women) of Bayesian marginal posterior distributions related to expressions of Lesion pattern #10: −0.30, 90% HPDI: −0.56 to −0.07, Fig. 3, c.f., Supplementary Figs 2 and 3 for an exhaustive display of difference distributions].

### Sensitivity analyses indicate stability of results

Total lesion volume, parcel-wise lesion volumes, as well as parcel-wise frequencies of lesion status did not significantly differ between men and women (*P* > 0.05, family wise error-corrected, Supplementary Table 2). Results remained unchanged after reiterating the identical analysis workflow in balanced groups of men and women with favourable outcomes: In the 20 logistic regression analyses resulting after downsampling, we consistently observed female-specific Lesion pattern #10 effects, i.e. the difference distribution of Lesion pattern #10 posteriors did not overlap with zero in 16 cases (80% of cases). In the remaining four downsampling scenarios, the difference distributions had a minor overlap with zero. Importantly, only the female-specific Lesion pattern #10 posterior distributions indicated a substantial effect, while the male-specific Lesion pattern #10 distributions overlapped with zero. Altogether, these downsampling analyses thus reinforced the notion that detected sex differences were not due to imbalances in the men:women ratio or more favourable long-term outcomes in men.

### Pronounced lesion connectivity disturbances in women in Lesion pattern #10

**Figure 4:**
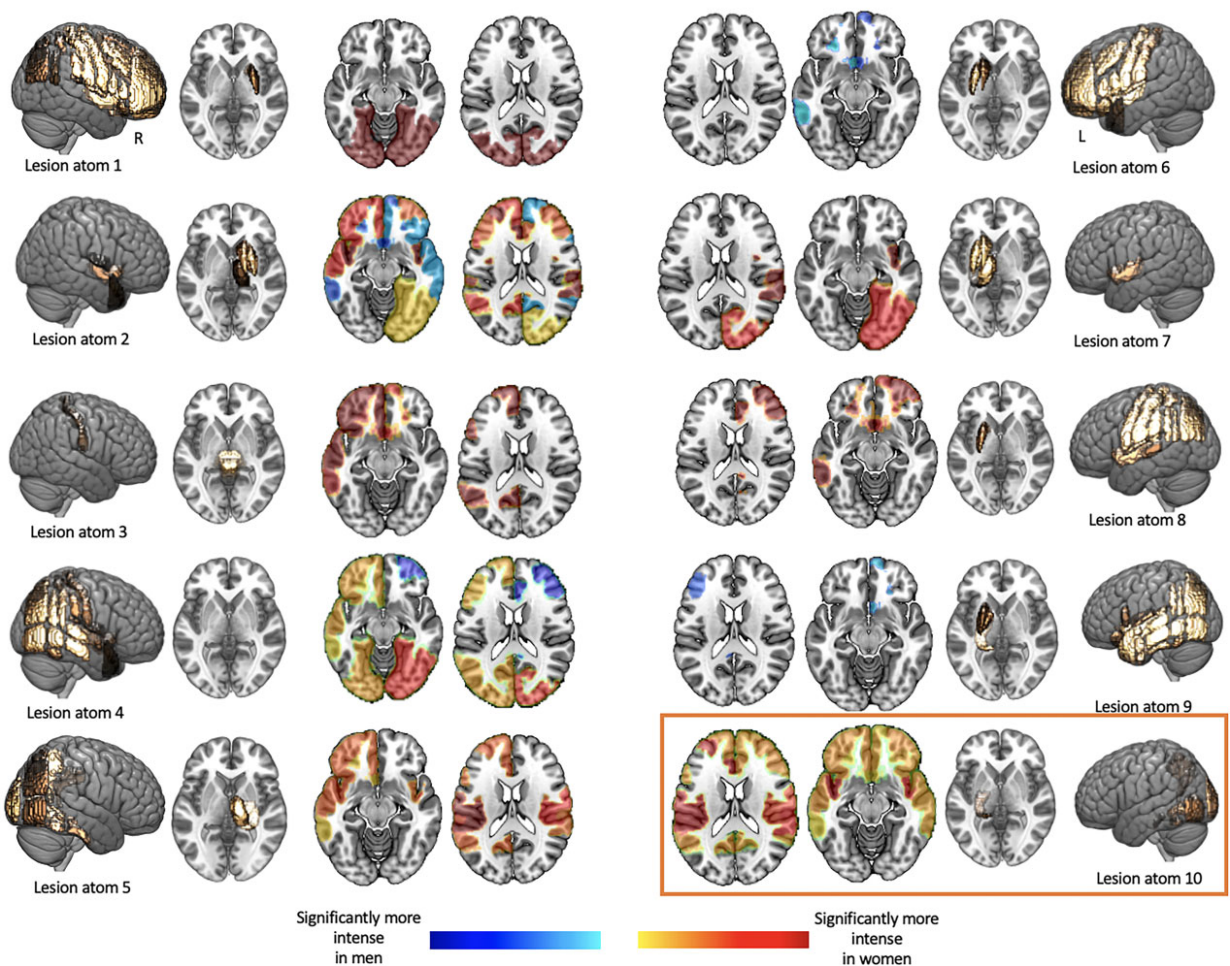
**Significantly altered male- and female-connectome-based lesion connectivity in 14 cortical networks (seven per hemisphere)**. Sex-specific lesion connectivity was computed for each of the 10 unique lesion patterns (c.f., Fig. 1) and subsequently statistically compared within each of the cortical networks. Networks with significantly different lesion connectivity are represented in colour (orange/red: significantly stronger in women; blue: significantly stronger in men). To allow for these statistical group comparisons in the first place, we inserted an additional simulation step: each lesion pattern was slightly varied 100 times by sampling a random number and collection of lesion pattern-affiliated brain regions. Exemplarily more in detail: Lesion pattern #1 primarily comprised the parcels pre- and post-central gyrus, superior, middle or inferior frontal gyrus, insular cortex, superior parietal and supramarginal cortex. We would here, for example, randomly choose pre- and post-central gyrus for a first Lesion pattern #1-like lesion, subsequently choose superior parietal, as well as supramarginal cortex for a second Lesion pattern #1-like lesion and so forth until we obtained 100 Lesion pattern #1-like lesions. Male- and female-specific lesion connectivity was then computed for each of these 100 simulated lesions per lesion pattern. Lesion patterns #2 and #10 comprised most connectivity differences (nine each). While Lesion pattern #2 was characterized by both higher connectivity in men and women, Lesion pattern #10 featured higher lesion connectivity exclusively in women.

Lesion pattern #10 was characterized by the most pronounced differences in lesion connectivity as uncovered by LNM (Fig. 4). More specifically, Lesion pattern #10 featured nine significant male- and female-specific intensity differences, that were consistent with women expressing more intense lesion connectivity. Only one other lesion pattern (Lesion pattern #2) had the same number of significant sex-specific intensity differences. Here, higher intensity however occurred for male- and female-based connectivity. Altogether, these explorations thus suggest that sex differences, as ascertained for Lesion pattern #10, might be linked to differences in affected functional connectivity. In women, Lesion pattern #10 was associated with a more pronounced deterioration in functional connectivity than in men.

## Discussion

Our study provides evidence that left-lateralized posterior circulation strokes increase the likelihood of unfavourable 3-month outcomes specifically in women. This effect was not found in male AIS patients. Our present findings thereby support and extend previous inferences on sex-specific lesion topographies for acute stroke severity^4^: lesions affecting the left hippocampus, precuneus, lingual and fusiform gyrus, the occipital pole and latero-occipital cortex, do not only explain more severe strokes in women in the acute phase. Rather, they have an additional permanent female-specific effect and explain unfavourable outcomes in a later phase after stroke. Future studies are warranted to now test whether women with these brain regions at risk may benefit from more aggressive acute reperfusion treatments, special post-acute rehabilitative efforts and a sex-specific planning of care.

Sex divergences in acute symptoms and long-term outcomes post-stroke are frequently examined with respect to sociodemographic and clinical aspects: For example, previous studies have suggested that women experience a higher stroke severity partly due to their more advanced age and higher likelihood of pre-morbid disability, as well as higher frequency of atrial fibrillation and cardioembolic strokes.^2,21^ However, the importance of actual lesion characteristics has rarely been studied in a sex-specific manner, that is lesion effects in men and women have rarely been compared, neither in low dimensions (e.g. total lesion volume) nor in high-dimensional settings (e.g. spatially high-resolution lesion-symptom mapping studies).^22^ In most cases, if considered at all, the biological sex of patients is regressed out from lesion information,^23^ essentially rendering it impossible to subsequently deduce any linear sex-specific effects. In contrast, the here installed Bayesian hierarchical modelling framework allowed for the estimation and systematic comparison of male- and female-specific lesion effects. Our findings enabled by these sex-specific pattern analyses now (i) offer the potential to enhance stroke outcome predictions and (ii) motivate the focused investigation of sex-stratified stroke care decisions with the ultimate goal of improving long-term outcomes.

Neuroanatomically, it is particularly striking that observed sex differences pertained to the *left* hemisphere only. Rare examples of early sex-specific lesion studies already indicated that left-sided lesions may have comparatively more detrimental effects in women.^24,25^ These studies contrasted left- and right-hemispheric strokes and their effects on intelligence measures in men and women. They concluded that women experience deterioration of both verbal and performance scale IQ values after left-hemispheric lesions, while in men and in the case of right-hemispheric lesions just one of these qualities was affected.^24,25^ Additionally, a recent functional connectivity-based study on sex-stratified predictions of IQ scores reported a high predictive capacity of *fusiform gyrus*-related functional connectivity patterns specifically in women.^26^ Of note, the left fusiform gyrus comprises the ‘visual word form area’ that is thought to be involved in the identification of words from their visual shape and thus to contribute to high-level processing of word meaning.^27^ Collectively, these previous insights may motivate the hypothesis that the left occipital fusiform gyrus, as encompassed in our female-specific left posterior circulation lesion pattern, could be one of the driving forces for our observed sex divergences. Nonetheless, a direct comparison of these earlier findings and ours is hampered by the crudeness of our spatial lesion representation and choice of outcome score.

Therefore, the coarse-grained nature of our 10 lesion patterns, that each combined several individual brain regions, as well as of our global 3-month clinical endpoint, unfavourable outcome as measured on the mRS, can be considered limitations of this study. Nonetheless, the spatial granularity of our lesion patterns goes beyond one of the early intelligence-focused lesion studies that differentiated between left- and right-hemispheric strokes only.^24,25^ Anatomical configurations of stroke lesions are furthermore naturally constrained by the human vasculature and our data-driven dimensionality reduction approach addresses this circumstance overtly by combining brain regions that tend to be lesioned simultaneously. Furthermore, the here-derived low-dimensional lesion representations have been shown to be highly reproducible in independent datasets in previous work.^4^ The mRS captures a patient’s functional status on a very general level only, but is frequently used as a primary outcome measure in large clinical stroke trials.^28^ It may thus represent a natural first choice; especially, as ascertaining sex differences in lesion topographies for such a global outcome automatically suggests a meaningful impact on a broad stroke population basis. Future studies are, however, needed to pinpoint the exact brain correlates and brain functions of the detected female-specific effects and to furthermore disentangle the effects of pre-stroke functional states and acute recanalization therapies. For example, a concrete first step could be to conduct prospective stroke trials to systematically evaluate sex-specific treatment decisions, such as more aggressive endovascular reperfusion therapies of the posterior cerebral artery (PCA) occlusions in women. Especially since thrombectomy for primary distal PCA occlusion stroke was recently determined to be safe and potentially beneficial,^29^ we would hypothesize a resulting improvement in outcomes in view of our present findings. Importantly, posterior circulation strokes occur in ∼30% of all cases across both men and women^30^ and approximately 13% of all strokes more specifically relate to PCA occlusions,^31^ underscoring the potential benefit of enhanced sex-specific treatment outcomes. Finally, our performance estimates were computed based on the Bayesian posterior predictive distributions, as analyses were performed in a Bayesian hierarchical regression framework. Especially if future work shifted the focus from inference on sex differences, as applied here, to a focus on clinical outcome prediction,^32,33^ this future work could complement our work by running cross-validated out-of-sample predictions. We here aimed to mitigate the risk of overfitting by investigating a limited number of input variables in a large sample of ∼800 stroke patients. The Bayesian priors may moreover intrinsically exert some regularizing effect.

Our study brings into sharp focus the possibility that lesions in the left posterior circulation underlie a female-specific higher likelihood of unfavourable functional outcomes approximately 3 months after AIS. Of note, we did not observe any sex differences in lesion volumes per se and findings remained the same when controlling for varying rates in unfavourable outcomes between men and women. If confirmed in future research, these findings prompt a more sex-specific approach to the planning of acute stroke trials in the shorter and clinical stroke management in the longer term. Our findings suggest that women could, exemplarily, benefit from more aggressive acute treatments of distal PCA occlusions. These more sex-informed clinical practices may eventually augment AIS outcomes on a broad population basis.

## Supplementary Material

fcac020_Supplementary_Data

## Appendix I

### MRI-GENIE and GISCOME Investigators and the International Stroke Genetics Consortium

Anna K. Bonkhoff, Martin Bretzner, Sungmin Hong, Markus D. Schirmer, Alexander Cohen, Robert W. Regenhardt, Kathleen L. Donahue, Marco J. Nardin, Adrian V. Dalca, Anne-Katrin Giese, Mark R. Etherton, Brandon L. Hancock, Steven J. T. Mocking, Elissa C. McIntosh, John Attia, Oscar R. Benavente, Stephen Bevan, John W. Cole, Amanda Donatti, Christoph J. Griessenauer, Laura Heitsch, Lukas Holmegaard, Katarina Jood, Jordi Jimenez-Conde, Steven J. Kittner, Robin Lemmens, Christopher R. Levi, Caitrin W. McDonough, James F. Meschia, Chia-Ling Phuah, Arndt Rolfs, Stefan Ropele, Jonathan Rosand, Jaume Roquer, Tatjana Rundek, Ralph L. Sacco, Reinhold Schmidt, Pankaj Sharma, Agnieszka Slowik, Martin Söderholm, Alessandro Sousa, Tara M. Stanne, Daniel Strbian, Turgut Tatlisumak, Vincent Thijs, Achala Vagal, Johan Wasselius, Daniel Woo, Ramin Zand, Patrick F. McArdle, Bradford B. Worrall, Christina Jern, Arne G. Lindgren, Jane Maguire, Michael D. Fox, Danilo Bzdok, Ona Wu and Natalia S. Rost.

## Abbreviations

AIS =: acute ischaemic stroke
AUC =: area under the receiver operating characteristic curve
DWI =: diffusion-weighted imaging
HO =: Harvard–Oxford
HPDI =: highest probability density interval
LNM =: lesion network mapping
mRS =: modified Rankin Scale
NMF =: non-negative matrix factorization
PCA =: posterior cerebral artery
