# Supplementary material for: Sex-specific lesion pattern of functional outcomes after stroke
Source: Brain Commun. 2022 Feb 2;4(2):fcac020. doi: 10.1093/braincomms/fcac020 (PMC8914504; doi:10.1093/braincomms/fcac020)
Supplement: fcac020_Supplementary_Data [file fcac020_supplementary_data.pdf]

Supplemental materials

MRI-GENIE: Sample size calculation

A total of 2,765 MRI-GENIE patients had automatically segmented DWI-defined stroke lesions.[2] Out of these, 1,920 (70.1%) were of sufficient quality and hence passed internal quality control by two experienced raters (M.B., A.K.B.). The final number of 822 included subjects was determined based on the additional availability of information on covariates and ~3 months mRS outcomes.

Supplementary Table 1. Sample sizes and characteristics of individual sites.

	BASICMAR (Spain)	SAHLSIS/GOTEBU (Sweden)	LEUVEN (Belgium)	GCNKSS (USA)	GASROS (USA)
**No. of subjects**	62	111	268	121	260
**Age**	70.1 (10.3)	51.5 (12.3)	67.0 (14.8)	67.6 (13.5)	65.2 (14.8)
**Sex**	33.9% female	30.6% female	41.4% female	44.6% female	39% female
**Modified Rankin Scale**	2 (2)	1 (1)	1 (1)	2 (2)	1 (1)
**Unfavorable outcomes**	41.9%	17.1%	24.3%	42.2%	25.8%

Neuroimaging parameters

Images were obtained on either 1T, 1.5T or 3T scanners (General Electric Medical Systems, Philips Medical Systems, Siemens, Toshiba, Marconi Medical Systems, Picker International, Inc.).

Diffusion-weighted images (DWI): Mostly axial scans (2727/2770 axial, 43/2770 coronal). Axial: Reconstruction matrix: mainly 256x256mm^2 (range: 128x128 to 432x384mm^2). Median field-of-view: 230 mm (range: 200 to 420 mm). Median slice thickness: 5mm (range: 2 to 7mm, gaps: 0 to 3mm). Median TR: 4.773ms, median TE: 92ms. Coronal: median TR: 8.200ms and median TE: 112ms, 5mm thickness, field-of-view 260mm, reconstruction matrix 256x256 mm^2. Most sequences: 3 directions (range: 3 to 25), one low b-value: 0s/mm^2 (range: 0 to 50s/mm^2), one high b-value: 1000s/mm^2 (range: 800 to 2000s/mm^2).

Full Bayesian hierarchical model specifications

Hyperpriors

$$hyper\_\sigma\_\beta \sim Halfcauchy(1)$$

$$\sigma\_\beta_{m,f} \sim Halfcauchy(hyper\_\sigma\_\beta)$$

$$hyper\_mu\_\beta \sim Normal(\mu = 0, \sigma = 1)$$

$$mu\_\beta_{m,f} \sim Normal(\mu = hyper\_mu\_\beta, \sigma = 1)$$

Priors

$$\alpha \sim Normal(\mu = 0, \sigma = 1)$$

$$\beta_{1\text{-}10;\, m,f} \sim Normal(\mu = mu\_\beta_{m,f}, \sigma = \sigma\_\beta_{m,f})$$

$$\beta_{age} \sim Normal(\mu = 0 , \sigma = 1)$$

$$\beta_{age*age} \sim Normal(\mu = 0 , \sigma = 1)$$

$$\beta_{sex} \sim Normal(\mu = 0 , \sigma = 1)$$

$$\beta_{hypertension} \sim Normal(\mu = 0 , \sigma = 1)$$

$$\beta_{diabetes} \sim Normal(\mu = 0 , \sigma = 1)$$

$$\beta_{atrial\ fibrillation} \sim Normal(\mu = 0 , \sigma = 1)$$

$$\beta_{coronary\ artery\ disease} \sim Normal(\mu = 0 , \sigma = 1)$$

$$\beta_{coronary\ artery\ disease} \sim Normal(\mu = 0 , \sigma = 1)$$

$$\beta_{prior\ stroke} \sim Normal(\mu = 0 , \sigma = 1)$$

$$\beta_{lesion\ volume} \sim Normal(\mu = 0 , \sigma = 1)$$

Linear model

$$mRS\_bin\_est = \alpha + \beta_{1\text{-}10}\,[sex] * NMF\text{-}Component_{1\text{-}10} + \beta_{age} * Age\ \beta_{age*age} * Age^{2} + \beta_{sex} * Sex + \beta_{hypertension} * \text{hypertension} + \beta_{diabetes} * \text{diabetes} + \beta_{atrial\ fibrillation} * \text{atrial fibrillation} + \beta_{coronary\ artery\ disease} * \text{coronary artery disease} + \beta_{prior\ stroke} * \text{prior stroke} + \beta_{lesion\ volume} * \text{Lesion volume}$$

Model likelihood

$$Unfavorable\ functional\ outcome \sim Bernoulli(p = deterministic\_sigmoid(mRS\_bin\_est))$$

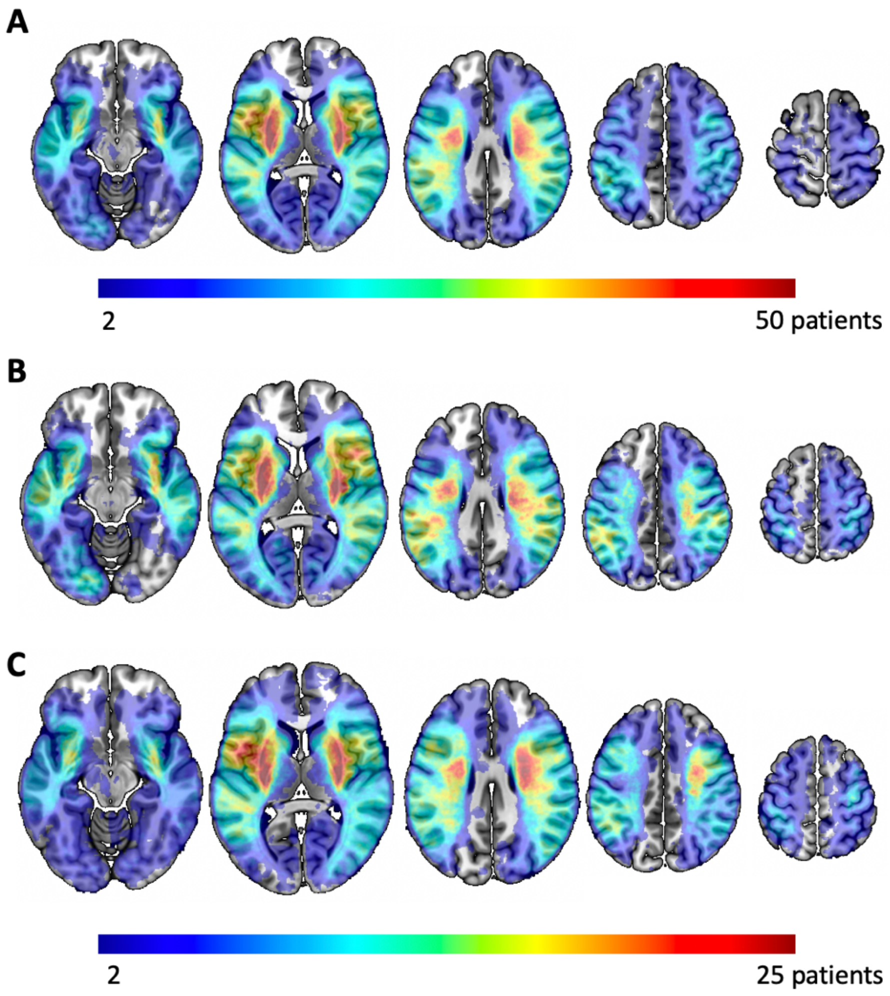

Supplementary Figure 1. Lesion overlaps. A Lesion overlap of all 822 male and female patients. **B** Lesion overlap of all 500 male patients. **C** Lesion overlap of all 322 female patients.

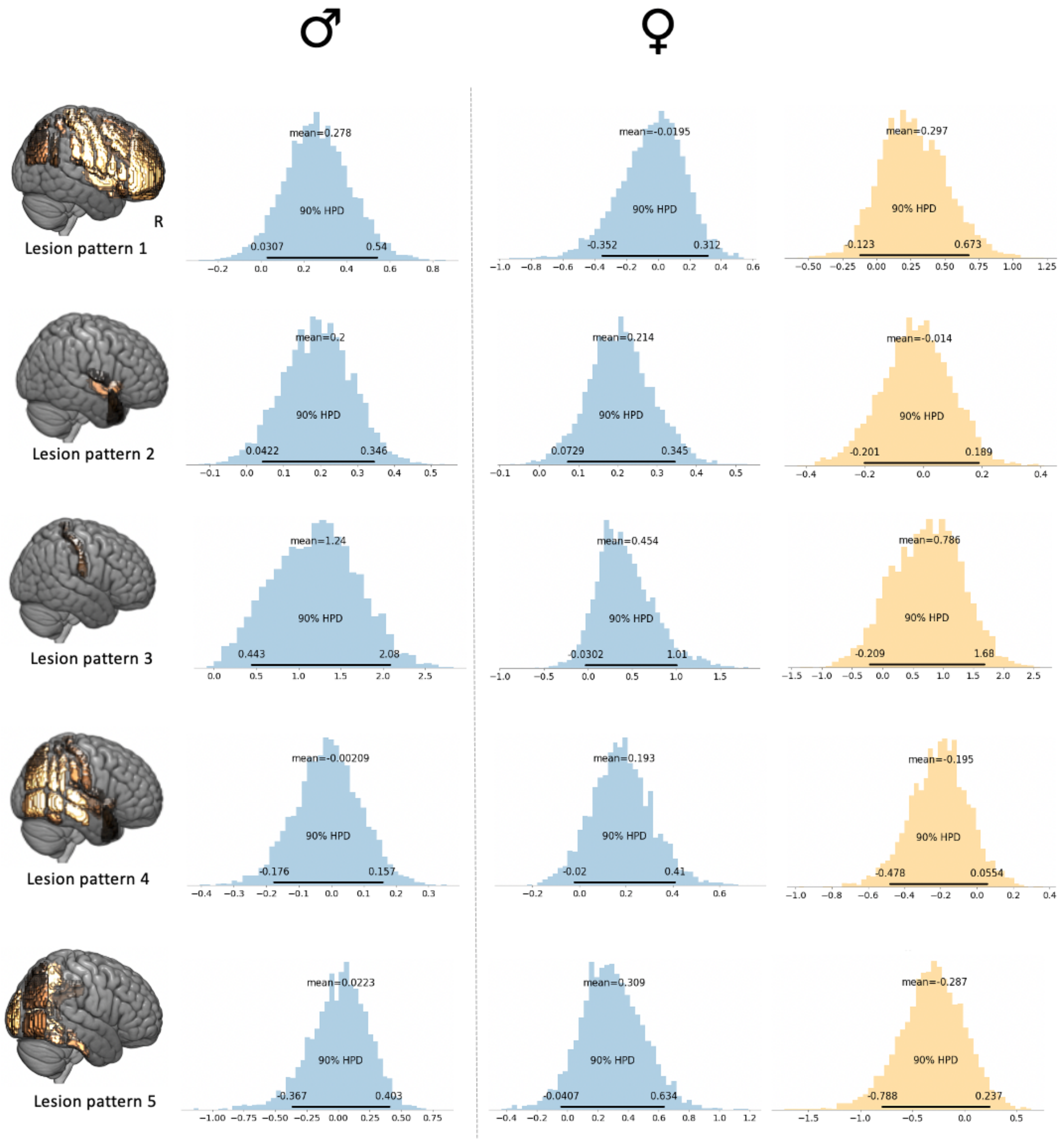

Supplementary Figure 2. Right-hemispheric sex-specific lesion pattern effects on long-term functional outcomes. From left to right: Renderings of structural lesion pattern configurations, male-specific posterior distributions of lesion pattern effects, female-specific posterior distributions of lesion pattern effects and finally their comparison. We assumed noteworthy lesion pattern effects or sex differences if 90% highest probability density intervals (HPDI) of sex-specific posterior or difference distributions did not overlap with zero. There were no substantial sex differences in case of right-hemispheric lesions. However, posterior distributions for lesion

pattern #2 did not substantially overlap with zero for both men and women; indicating a consistent effect for both biological sexes.

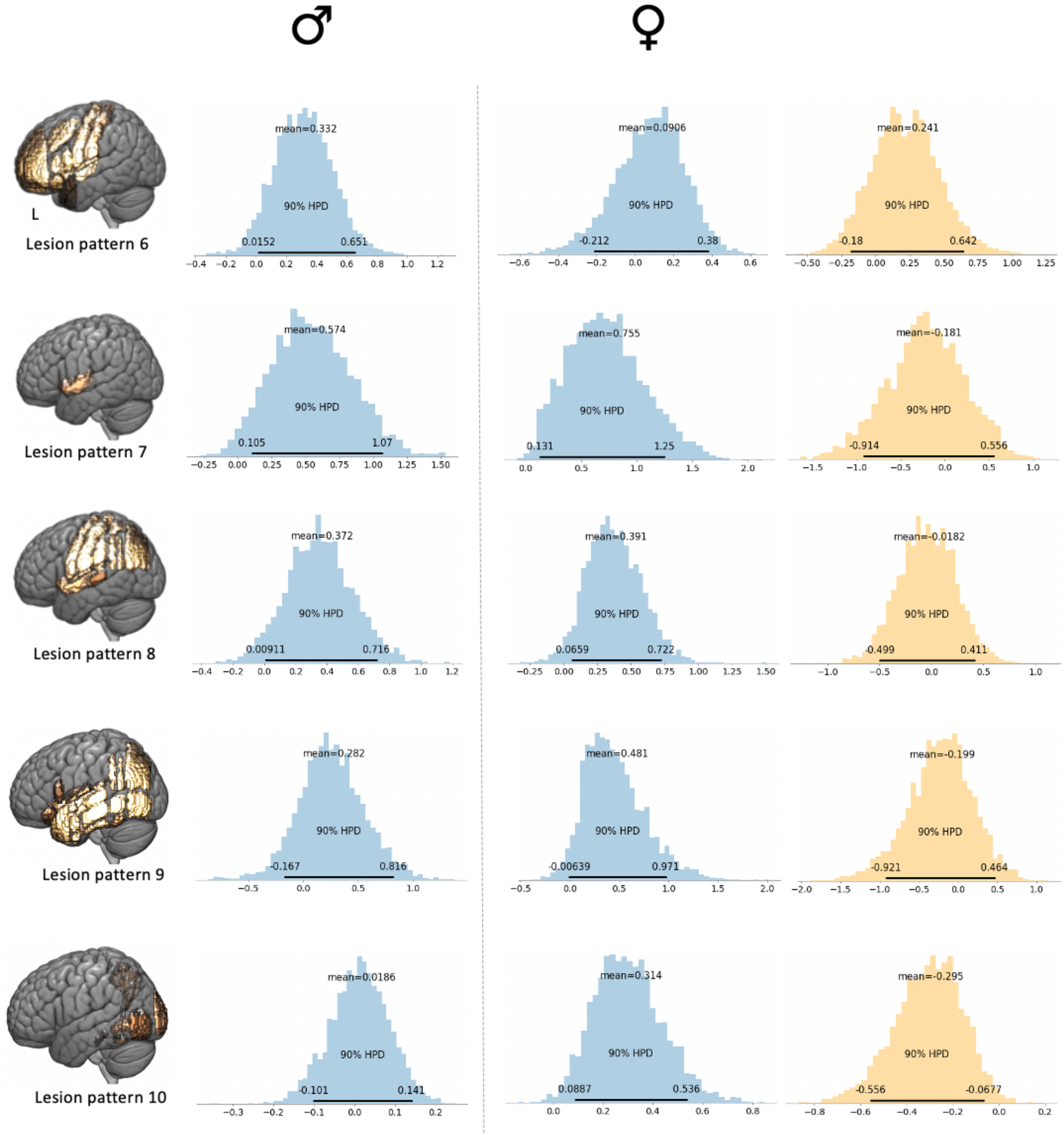

Supplementary Figure 3. Left-hemispheric sex-specific lesion pattern effects on long-term functional outcomes. As also outlined for **Supplementary Figure 2**: Renderings of structural lesion pattern configurations are visualized on the left. Next, male-specific posterior distributions of lesion pattern effects and female-specific posterior distributions of lesion pattern effects are displayed. Their comparison is highlighted on the right. Lesion patterns #7 and #8 have substantial effects across men and women. Lesion pattern #10 comprised a substantial sex difference, as inferable from the difference distribution that does not meaningfully overlap with zero.

Supplementary Table 2: Region-wise lesion loads and frequencies: Women versus Men. Sex differences in parcel-wise lesion volumes and frequencies of how often a specific parcel was affected were examined via independent two-sample t-tests and Fisher's exact tests (level of significance: $p<0.05$, FEW-corrected for multiple comparisons).

**Region**	**Region-wise lesion load: *p*-value (Bonferroni-corrected)**	**Region-wise frequencies: *p*-value (Bonferroni-corrected)**
Left Frontal Pole	1	1
Right Frontal Pole	1	1
Left Insular Cortex	1	1
Right Insular Cortex	1	1
Left Superior Frontal Gyrus	1	1
Right Superior Frontal Gyrus	1	1
Left Middle Frontal Gyrus	1	1
Right Middle Frontal Gyrus	1	1

Left Inferior Frontal Gyrus, pars triangularis	1	1
Right Inferior Frontal Gyrus, pars triangularis	1	1
Left Inferior Frontal Gyrus, pars opercularis	1	1
Right Inferior Frontal Gyrus, pars opercularis	1	1
Left Precentral Gyrus	1	1
Right Precentral Gyrus	1	1
Left Temporal Pole	1	1
Right Temporal Pole	1	1
Left Superior Temporal Gyrus, anterior division	1	1
Right Superior Temporal Gyrus, anterior division	1	1
Left Superior Temporal Gyrus, posterior division	1	1
Right Superior Temporal Gyrus, posterior division	1	1

Left Middle Temporal Gyrus, anterior division	1	1
Right Middle Temporal Gyrus, anterior division	1	1
Left Middle Temporal Gyrus, posterior division	1	1
Right Middle Temporal Gyrus, posterior division	1	1
Left Middle Temporal Gyrus, temporooccipital part	1	1
Right Middle Temporal Gyrus, temporooccipital part	1	1
Left Inferior Temporal Gyrus, anterior division	1	1
Right Inferior Temporal Gyrus, anterior division	1	1
Left Inferior Temporal Gyrus, posterior division	1	1
Right Inferior Temporal Gyrus, posterior division	1	1

Left Inferior Temporal Gyrus, temporooccipital part	1	1
Right Inferior Temporal Gyrus, temporooccipital part	1	1
Left Postcentral Gyrus	1	1
Right Postcentral Gyrus	1	1
Left Superior Parietal Lobule	1	1
Right Superior Parietal Lobule	1	1
Left Supramarginal Gyrus, anterior division	1	1
Right Supramarginal Gyrus, anterior division	1	1
Left Supramarginal Gyrus, posterior division	1	1
Right Supramarginal Gyrus, posterior division	1	1
Left Angular Gyrus	1	1
Right Angular Gyrus	1	1

Left Lateral Occipital Cortex, superior division	1	1
Right Lateral Occipital Cortex, superior division	1	1
Left Lateral Occipital Cortex, inferior division	1	1
Right Lateral Occipital Cortex, inferior division	1	1
Left Intracalcarine Cortex	1	1
Right Intracalcarine Cortex	1	1
Left Frontal Medial Cortex	1	1
Right Frontal Medial Cortex	1	1
Left Supplementary Motor Cortex	1	1
Right Supplementary Motor Cortex	1	1
Left Subcallosal Cortex	1	1
Right Subcallosal Cortex	1	1
Left Paracingulate Gyrus	1	1

Right Paracingulate Gyrus	1	1
Left Cingulate Gyrus, anterior division	1	1
Right Cingulate Gyrus, anterior division	1	1
Left Cingulate Gyrus, posterior division	1	1
Right Cingulate Gyrus, posterior division	1	1
Left Precuneous Cortex	1	1
Right Precuneous Cortex	1	1
Left Cuneal Cortex	1	1
Right Cuneal Cortex	1	1
Left Frontal Orbital Cortex	1	1
Right Frontal Orbital Cortex	1	1
Left Parahippocampal Gyrus, anterior division	1	1
Right Parahippocampal Gyrus, anterior division	1	1

Left Parahippocampal Gyrus, posterior division	1	1
Right Parahippocampal Gyrus, posterior division	1	1
Left Lingual Gyrus	1	1
Right Lingual Gyrus	1	1
Left Temporal Fusiform Cortex, anterior division	1	1
Right Temporal Fusiform Cortex, anterior division	1	1
Left Temporal Fusiform Cortex, posterior division	1	1
Right Temporal Fusiform Cortex, posterior division	1	1
Left Temporal Occipital Fusiform Cortex	1	1
Right Temporal Occipital Fusiform Cortex	1	1
Left Occipital Fusiform Gyrus	1	1

Right Occipital Fusiform Gyrus	1	1
Left Frontal Operculum Cortex	1	1
Right Frontal Operculum Cortex	1	1
Left Central Opercular Cortex	1	1
Right Central Opercular Cortex	1	1
Left Parietal Operculum Cortex	1	1
Right Parietal Operculum Cortex	1	1
Left Planum Polare	1	1
Right Planum Polare	1	1
Left Heschl's Gyrus	1	1
Right Heschl's Gyrus	1	1
Left Planum Temporale	1	1
Right Planum Temporale	1	1
Left Occipital Pole	1	1
Right Occipital Pole	1	1
Left Thalamus	1	1
Left Caudate	1	1
Left Putamen	1	1
Left Pallidum	1	1
Brain-Stem	1	1
Left Hippocampus	1	1

Left Amygdala	1	1
Left Accumbens	1	1
Right Thalamus	1	1
Right Caudate	1	1
Right Putamen	1	1
Right Pallidum	1	1
Right Hippocampus	1	1
Right Amygdala	1	1
Right Accumbens	1	1
anterior thalamic radiation l	1	1
anterior thalamic radiation r	1	1
corticospinal tract l	0.63	1
corticospinal tract r	1	1
cingulum 1	1	1
cingulum 2	1	1
cingulum 3	1	1
cingulum 4	1	1
forceps major	1	1
forceps minor	1	1
inferior fronto occipital fasciculus l	1	1
inferior fronto occipital fasciculus r	1	1
inferior longitudinal fasciculus l	1	1
inferior longitudinal fasciculus r	1	1

superior longitudinal fasciculus l	1	1
superior longitudinal fasciculus r	1	1
uncinate fasciculus l	1	1
uncinate fasciculus r	1	1
superior longitudinal fasciculus (temp) l	1	1
superior longitudinal fasciculus (temp) r	1	1
